# Spatiotemporal distribution of *Escherichia coli* and fecal and non-fecal pathogens in the urban surface water system of Amsterdam

**DOI:** 10.1007/s10661-026-15653-y

**Published:** 2026-07-20

**Authors:** Sha Gao, Thomas Wagner, Paul van der Wielen, Huub Rijnaarts, Nicolae Șișcanu, Nora Sutton

**Affiliations:** 1 Department of Environmental Technology, Wageningen University, Wageningen, PO Box 17, 6700 EV The Netherlands; 2 KWR Water Research Institute, Groningenhaven 7, Nieuwegein, 3433PE The Netherlands

**Keywords:** Microbial water quality, Monitoring, Urban surface water, Fecal pathogen, Non-fecal pathogen, Fecal indicator bacteria

## Abstract

Understanding the microbial quality of urban surface water, particularly pathogens dynamics, is crucial for determining public health risks from recreation and for developing effective monitoring strategies in water-centric cities like Amsterdam. We monitored six locations along Amstel River and inner canals between May and October 2023 using ultrafiltration sampling and qPCR to quantify *Escherichia coli*, four fecal, and three non-fecal pathogens. Spearman’s correlations were conducted to explore relationships between *E. coli*, pathogens, water temperature, and rainfall. *E. coli* and *Campylobacter* were consistently detected at all locations (2.1–5.2 and 1.7–6.4 log_10_ copies/100 mL), with higher *Campylobacter* levels in inner canals than Amstel River. Human adenovirus appeared in 17 of 36 samples (0.2–2.6 log_10_ copies/100 mL), most frequently near the wastewater treatment plant (WWTP) and correlated with rainfall (*p* < 0.01), suggesting runoff or effluent influences. *Cryptosporidium* and *Giardia* were each detected once. *Pseudomonas aeruginosa* was widespread (30/36 samples, 1.1–2.7 log_10_ copies/100 mL) without clear temporal or spatial patterns. *Legionella pneumophila* (14/36 samples, 2.1–3.5 log_10_ copies/100 mL) was more abundant near WWTP, indicating potential effluent effects. *Leptospira* occurred mainly in inner canals during May (0.3–1.8 log_10_ copies/100 mL), reflecting seasonal and spatial variability. Besides *Campylobacter* (*p* < 0.01), no pathogens showed significant correlations with *E. coli*, reinforcing its limitations as an indicator for fecal and the non-fecal pathogens in Amsterdam surface water system. Overall, pathogen occurrence showed spatiotemporal variation influenced by potential sources and weather conditions. Future monitoring frameworks should integrate selected fecal and non-fecal pathogens alongside traditional indicators to improve assessments of urban microbial water quality. Incorporating microbial source tracking markers may further enhance source attribution while balancing feasibility and cost.

**Supplementary Information:**

The online version contains supplementary material available at 10.1007/s10661-026-15653-y.

## Introduction

Ensuring safe microbial surface water quality is a global priority, as it is crucial for reducing health risks with recreational activities in and around surface water, such as swimming, fishing and sailing (Tiwari et al., 2021). The microbial surface water quality can be compromised by pathogenic microorganisms that pose a potential health risk. These pathogens can be divided based on their origin into fecal pathogens from animals and humans, such as *Campylobacter*,* Salmonella*,* Cryptosporidium*,* Giardia* and adenovirus, and non-fecal pathogens which naturally thrive in water, such as *P. aeruginosa*,* L. pneumophila*, and *Leptospira* (Tiwari et al., 2021). As a result of the impact of the microbial water quality on the open water swimming and triathlon competition during the 2024 Olympic Games in Paris, the microbial urban surface water quality has attracted worldwide attention. In recent years, many European cities have initiated efforts to improve the water quality in their urban waterways, with Amsterdam, Copenhagen, Vienna, Paris, and Berlin leading the way (Wright, 2024).

In Amsterdam and surroundings, several official swimming locations have been designated and are under rigorous water quality monitoring (Peters et al., 2021; Waternet, 2015). Additionally, emergency measures are in place for swimming events, such as the Amsterdam City Swim in the canals, to limit the health risks, including intensive monitoring of the microbial water quality and managing contamination sources by e.g. monitoring sewer overflow nearby the swimming route. Despite these efforts, health complaints have been reported; for instance, 31% of the participants in the 2015 edition reported suffering from gastroenteritis after the event, likely caused by norovirus (Joosten et al., 2017). The potential sources of fecal contamination in Amsterdam’s rivers and canals have been identified, namely, excrements of flying birds and waterfowls, combined sewer overflow as a result of excessive rainfall, street dirt migrating with surface runoff, and the incidental wastewater discharge from the thousands of houseboats (which are mostly connected to the sewage system but some have damaged or leaking pipes) (Kusumawardhana et al., 2021; Sales-Ortells & Medema, 2014; Sales-Ortells et al., 2015). A health risk assessment for various water-based recreational activities in Amsterdam indicated a high probability of gastrointestinal illness (GI) for those engaged in activities, such as playing in Wadi and pluvial floods from combined sewer overflows and swimming or rowing in urban rivers as a result of exposure to *Campylobacter* (Sales-Ortells & Medema, 2014; Sales-Ortells et al., 2015)*.*

In addition to GI risks from fecal pathogens, high concentrations of non-fecal pathogens have also been reported in surface waters in the Netherlands. For example, *L. pneumophila*, the most virulent *Legionella* species responsible for 90–95% of all reported *Legionella* cases in the Netherlands, was detected at a relatively high concentration of up to 1.3 × 10^3^ gc/100 mL in a rainwater collection pond in Amsterdam (Sales-Ortells et al., 2015). In addition, 97 human leptospirosis cases, caused by *Leptospira*, in the Netherlands were reported in 2014 from both autochthonous (*n* = 60) and imported cases (*n* = 37) (Pijnacker et al., 2016). About 37 of the autochthonous cases were attributed to surface water contact, including recreational exposure, and 13 cases from direct contact with mainly rats (Pijnacker et al., 2016). Furthermore, frequent outbreaks of ear infections during swimming activities in the Netherlands have been attributed to *P. aeruginosa* (Schets et al., 2011a, 2011b). Although non-fecal pathogens contribute significantly to illness relating to waterborne pathogens (Collier et al., 2021), only fecal indicator bacteria (FIB), such as intestinal enterococci and *Escherichia coli*, are generally used to evaluate the microbial quality for bathing water according to the WHO and European Union bathing water regulations (WHO, 2003; European Parliament and Council of the European Union, 2006). An ideal microbial water quality indicator should be strongly associated with human health risk that relates to fecal pathogen and environmental opportunistic pathogens, exhibit environmental persistence comparable to relevant pathogens, not proliferate substantially in environmental waters, and be detectable using reliable and reproducible analytical methods (Harwood et al., 2014). However, these characteristics are not necessarily representative of non-fecal pathogens, many of which can survive and proliferate under favorable environmental conditions, thereby increasing the potential for severe disease outbreaks (Tiwari et al., 2021). Hence, a more thorough evaluation of risks of non-fecal pathogens in urban surface waters is needed, especially since water temperatures in more moderate climate zones are increasing due to climate change and which can enhance growth of these non-fecal pathogens (van der Wielen et al., 2023).

Previous research has explored the influence of climate conditions, such as rainfall, temperature and seasonal variation across different geographical regions, on FIB, source tracking markers, and gastroenteritis-causing pathogens like *Giardia* spp., *Cryptosporidium* spp., *Campylobacter*, and adenovirus (Carter et al., 1987; Hörman et al., 2004; Islam et al., 2017; Rodrigues et al., 2015; Victoria et al., 2014)*.* These studies have focused on the seasonal variations in concentrations and detection frequencies (occurrence/prevalence) of either individual or multiple gastroenteritis pathogenic bacteria, viruses, and/or protozoa in surface water and their correlation to FIB (Carter et al., 1987; Hörman et al., 2004; Silva et al., 2011), or on the influence of rainfall and temperature on FIB concentrations and associated health risks from gastroenteric viruses in city rivers, recreational beaches and basins (Islam et al., 2017; Rodrigues et al., 2015; Victoria et al., 2014). Another study showed that seasonal variation and land use were major factors affecting the occurrence and concentration patterns of FIB, gastroenteritis pathogenic bacteria, and protozoa, with FIB being reliable indicators for gastroenteritis pathogenic bacteria but not protozoa (Duris et al., 2013). These studies emphasize the substantial influence of climate conditions, seasonal variations, and land use on the distribution of fecal indicator bacteria (FIB), gastroenteritis-causing pathogens, and protozoa in surface waters.

Research on the spatial concentration patterns across environments or locations and occurrence of non-fecal pathogens has primarily focused on areas where relevant disease outbreaks have been reported. In the Netherlands, for example, legionellosis, ear infections, and leptospirosis caused by exposure to *L. pneumophila*,* P. aeruginosa*, and *Leptospira* are common clinical diseases that can be associated to surface waters (Pijnacker et al., 2016; Wullings et al., 2009). Further attention is warranted in the Netherlands (as an example of moderate climate urbanized region) on non-fecal pathogens due to their wide distribution in environments (Bierque et al., 2020), their ability to multiply in water (Musaazi et al., 2023; Kuiper, 2006; van Hoof et al., 2014) and the increasing trend in infections caused by these organisms (Pijnacker et al., 2016).

Overall, previous studies predominantly focused on the factors influencing microbial water quality using FIB over extended time periods or on source tracking for individual pathogens. This study distinguishes itself by (i) simultaneously quantifying fecal and non-fecal pathogens, (ii) applying large-volume (50 L) sampling to improve detection sensitivity, (iii) assessing spatial and temporal dynamics along an urban river-to-canal transect under varying climatic conditions, and (iv) assessing whether *E. coli* is a reliable indicator for the presence of pathogens at the sampling scale and studied area in Amsterdam during spring, summer, and autumn.

## Material and methods

### Sampling locations and sampling

**Fig. 1:**
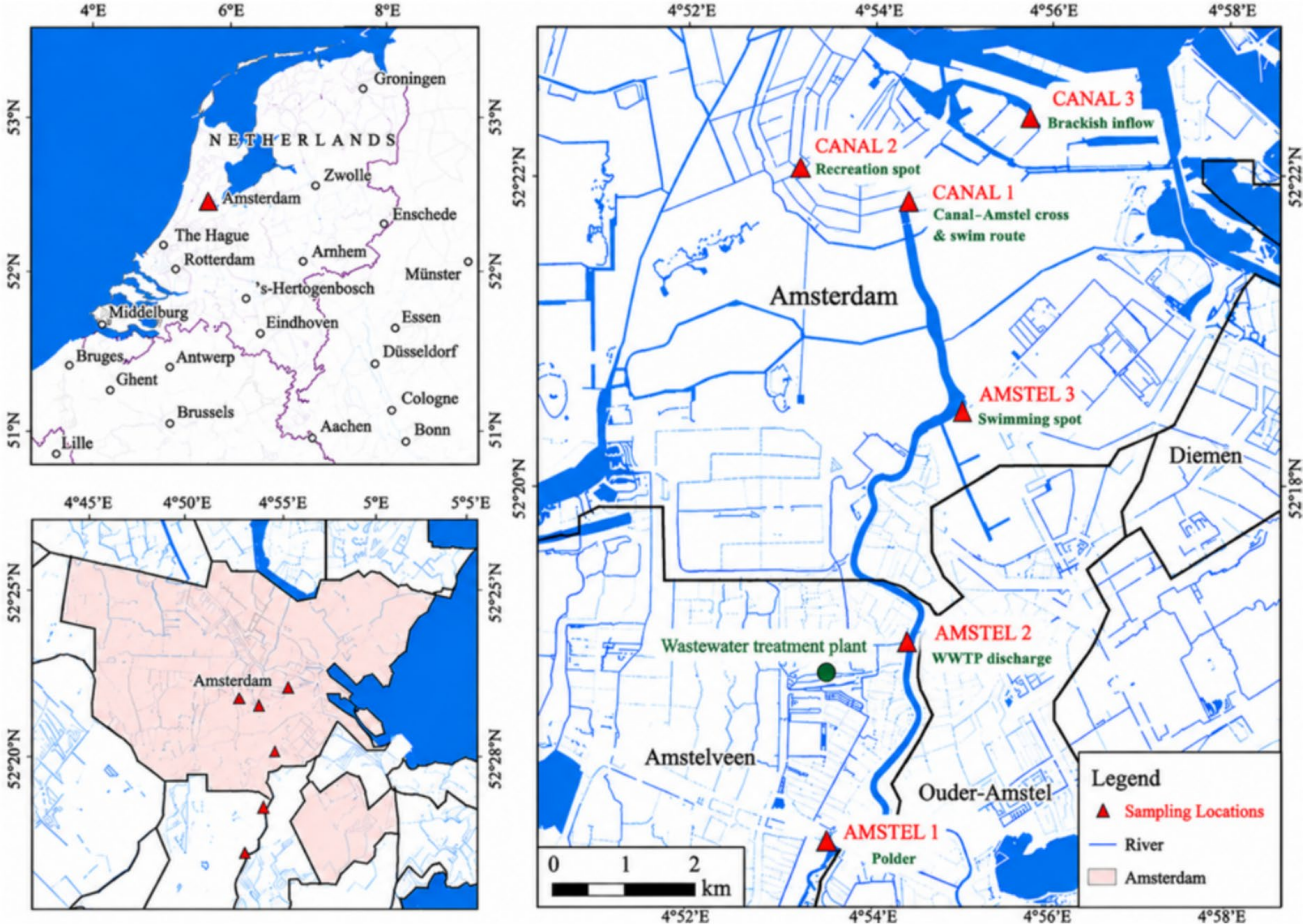
Map showing the six sampling locations in Amsterdam: AMSTEL 1, AMSTEL 2, AMSTEL 3, CANAL 1, CANAL 2, and CANAL 3

Samples for urban microbial surface water quality determination were obtained at six locations in Amsterdam: AMSTEL 1, AMSTEL 2, AMSTEL 3, CANAL 1, CANAL 2, and CANAL 3 (Fig. 1). These locations coincide with (microbial) water quality monitoring locations of the regional water authorities.

The locations are distributed along the Amstel River that flows from south to north through Amsterdam, passing the effluent discharge point of a WWTP, a sewer overflow area, and several places with frequent recreational activities (Fig. 1). AMSTEL 1 is located downstream the Rondehoep polder, where meadow birds reside and agricultural activities take place. Subsequently, AMSTEL 2 is located approximately 1 km downstream of the WWTP effluent discharge point. AMSTEL 3 is located at a location where a tributary river enters the Amstel River, in an area that is frequently used for swimming but also prone to sewer overflows (https://maps.amsterdam.nl/rainproof/). CANAL 1 is at the crossroad of the Amstel River and inner canals and is part of the route of the yearly Amsterdam City Swim event (Hintaran et al., 2018). CANAL 2 is located in the inner canals in an area where water recreation frequently occurs. Finally, CANAL 3 is located in the area where brackish water from the canal that connects the Amsterdam harbor with the North Sea enters the inner canals.

At each site, 50 L of water was collected in five of 10 L plastic jerry cans monthly between May and October for microbial analyses. Water temperature was measured on-site immediately after sampling. Sampling took 15–30 min per location, with a transport time of about 20 min between sites. Samples were transported at 4 °C to the laboratory (~ 80-min drive) and stored at 4 °C for later analysis.

### Selection of targeted pathogens

A number of fecal and non-fecal pathogens together with *E. coli* were selected as the monitoring targets in this study (Table 1). The selection was based on several publications regarding microbial water quality in the Netherlands (Sales-Ortells et al., 2015; Schets et al., 2008, 2011a, 2011b), resulting in a list of seven pathogens, which covers three categories of relevant waterborne pathogens (viruses, bacteria, and protozoa) and different possible illnesses (gastrointestinal; ear, eye, wound, and respiratory infections; skin irritation). *E. coli*, the fecal waterborne pathogens *Campylobacter*, adenovirus, *Cryptosporidium*, and *Giardia*, and the non-fecal waterborne pathogens *P. aeruginosa, Leptospira*, and *L. pneumophila* were monitored (Table 1).

**Table 1:** List of target pathogens and *E. coli* in this study

Category	Name	Origin	Related waterborne disease	Main transmission route
**Bacteria**	*E. coli*	Fecal indicator		
	*Campylobacter*	Human and animal feces	Gastroenteritis	Fecal-oral, through ingestion of contaminated water
	*Pseudomonas aeruginosa*	Non-fecal; water bodies	Infections in skin and ear (otitis externa) and pneumonia	Inhalation of contaminated water aerosols or skin-water contact
	*Leptospira*	Non-fecal; urine of rats, cattle, pigs and dogs	Leptospirosis	Ingestion of water contaminated with urine of the brown rat
	*Legionella pneumophila*	Non-fecal; water bodies	Legionnaires’ disease, Pontiac fever	Inhalation of contaminated water aerosols
**Protozoa**	*Cryptosporidium*	Human and animal feces	Cryptosporidiosis, gastroenteritis	Fecal–oral, through ingestion of contaminated water
	*Giardia*		Giardiasis, gastroenteritis	
**Virus**	Adenovirus (Ad40/41)	Human feces	Gastroenteritis, acute respiratory diseases, pneumonia	Inhalation of aerosolized droplets; fecal–oral, through ingestion of contaminated water

### Sample processing and analysis

#### Sample processing

**Fig. 2:**
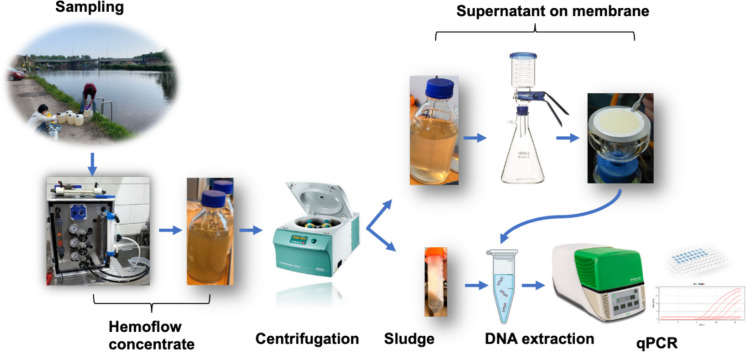
Schematic graph for samples processing

An NX Mexplorer nanofiltration test unit (NXFiltration, Hengelo, the Netherlands) was operated in crossflow filtration mode using a Hemoflow FX80 dialysis membrane (Fresenius Medical Care AG, Bad Homburg, Germany) and a 60 L FermZilla fermentation tank (Brewolution, Them, Denmark) to concentrate large volume water samples (Fig. 2). The Hemoflow FX80 membrane consists of a synthetic Helixone® polysulfone/polyvinylpyrrolidone (PVP) blend and has an effective surface area of 1.8 m^2^, an approximate molecular weight cut-off (MWCO) of 30–40 kDa, and an estimated effective pore size in the range of 3–10 nm. The 50 L samples were concentrated with this unit to approximately 0.7 L retentate volume at a transmembrane pressure of 1.2 bar and a flow rate of 4.2 L/min. Subsequently, 1 L of permeate/filtrate was recirculated through the system without additional pressure to rinse residual biomass from the tank walls and membrane tubing into the retentate fraction (Fig. 2). In a pre-test experiment, a reproducible recovery of 50% ± 7% for *E. coli* was obtained by the concentration procedure. Each sample was subsequently centrifuged at 9000 g for 10 min resulting in roughly 5 mL of pellet and 1.5 L supernatant fractions. The pellet was stored at −20 °C prior to DNA extraction. Subsequently, 100 mL aliquots of the supernatant were vacuum filtered in triplicate using 0.2 µm filters and filters were stored in Eppendorf 2 mL vials at −20 °C. The pellet and filter fractions were extracted and analyzed separately, and their quantified gene copy numbers were combined to calculate the final pathogen concentration (copies/100 mL) in the original water sample.

#### Microbiological analyses

DNA from 0.25 mL of the sludge samples was extracted in triplicate using the DNeasy PowerSoil Pro Kit (CAT NO./ID 47016, QIAGEN, Hilden, Germany) and eluted in 150 µL elution buffer. DNA from the 0.2 µm filters (containing the filtered supernatant fraction) was extracted in duplicate using the DNeasy PowerWater Kit (CAT NO./14900-50-NF, QIAGEN, Hilden, Germany) and eluted in 200 µL elution buffer. Negative extraction controls consisting of DNase/RNase-free water were included to monitor potential contamination during the extraction procedure. A plasmid containing dengue virus DNA fragments was used as an internal control (IC) to assess DNA extraction recovery and potential qPCR inhibition, as described previously (Sales-Ortells et al., 2015). Briefly, 10 µL of the IC was added during the first step of DNA extraction for each sample and quantified subsequently by qPCR. Recovery efficiencies based on the IC were included in the final concentration calculations.

Specific genes of the IC, *E. coli*,* L. pneumophila,* adenovirus 40/41, *Campylobacter* spp., *P. aeruginosa*,* Giardia assemblage A & B*,* Cryptosporidium*, and *Leptospira* in the DNA extracts were quantified using target-specific probe qPCR assays with serially diluted standard curves. The qPCR reaction mixture had a total volume of 25 µL and consisted of 12.5 µL iQ Supermix (Bio-Rad Laboratories B.V., Veenendaal, the Netherlands), 6.5 µL DNase-free water (Merck KGaA, Darmstadt, Germany), primers and probes at optimized concentrations, and 5 µL DNA extract. Primers, probes, and amplification conditions for each target were adopted from previously validated studies and are listed in the Supplementary Information (SI2, Table S1). Potential qPCR inhibition was evaluated using the IC amplification performance. Samples showing reduced amplification efficiency were diluted and reanalyzed when necessary. The qPCR reactions and calculations were performed using the CFX OPUS 96 system (Bio-Rad Laboratories B.V., Veenendaal, the Netherlands). Final concentrations of each organism in the original surface water sample were calculated as described in SI1.

#### Water temperature and rainfall

Water temperatures were measured in-situ at the sampling location immediately after the samples were taken with a Waterproof Conductivity Meter (Manutan BV, Den Dolder, Netherlands). Rainfall data in Amsterdam for the sampling dates were downloaded from a global weather forecast and history data collection website Visualcrossing (https://www.visualcrossing.com) (SI2, Table S2). The sum of rainfall in seven days prior to the sampling dates was used in this study as others reported that the influence of rainfall on pathogen concentrations last up to a week in the Amsterdam surface water system (Sales-Ortells et al., 2015).

### Statistical analysis

#### Correlation analysis

A correlation matrix between all parameters was calculated using IBM SPSS Statistics (version 28.0.0.0). The copy number concentrations of all pathogens were log10 transformed before analysis. A normality test was performed by Q-Q plots and Shapiro–Wilk descriptive analysis to confirm normality of the data. The results showed that the log10 transformed *E. coli* and *Campylobacter* data were normally distributed while the log10 transformed data of all other pathogens (*P. aeruginosa*,* L. pneumophila*, adenovirus, and *Leptospira*), the water temperature and the rainfall were not normally distributed. Therefore, the non-parametric Spearman correlation analysis with a confidence level of 99% (*p* < 0.01) was performed to determine significant correlations between pathogens, water temperature, and rainfall.

#### ANOVA analysis

To compare spatial and temporal differences in the log10 transformed concentrations of all microorganisms, water temperature, and rainfall (only temporally), a parametric one-way ANOVA with Bonferroni post hoc test or a non-parametric Kruskal–Wallis *H* test with a Bonferroni post hoc test was performed according to results of the normality test. The detailed results of *p* values and the type of test performed are listed in supporting information (Table S3). A significance level of *p* < 0.01 was applied for correlation analysis to reduce Type I error (i.e., false positive result), while *p* < 0.05 was used for ANOVA/Kruskal–Wallis tests following standard practice.

## Results

### Spatiotemporal distribution of *E. coli* and waterborne pathogens

#### Fecal microorganisms

**Fig. 3:**
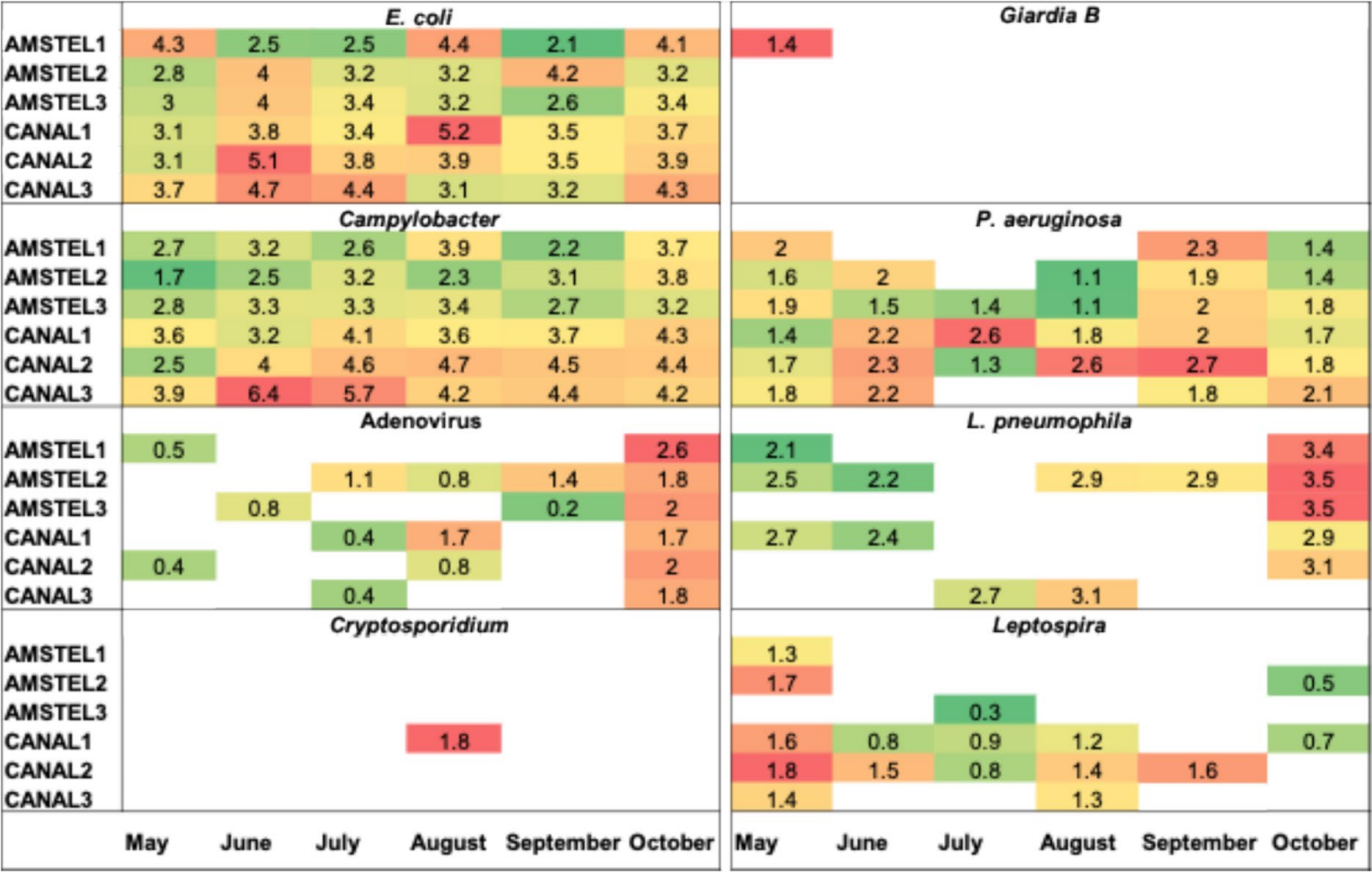
Heatmap of Log_10_ concentrations (copies/100mL) of *E. coli*,* Campylobacter*, adenovirus, *Cryptosporidium*,* Giardia*,* P. aeruginosa*,* L. pneumophila*, and *Leptospira* at all locations along the sampling time in 50-L water samples. Blank cells are below detection limit. Data are averages of duplicates

*E. coli* was consistently detected in concentrations ranging from 2.1 to 5.2 log_10_ copies/100 mL, across all sampling locations and times (Fig. 3). Concentrations exceeding 3.0 log_10_ copies/100 mL were always observed in the inner canals at locations CANAL 1, CANAL 2, and CANAL 3, whereas at the Amstel River locations, concentrations below 3.0 log_10_ copies/100 mL were observed as well. The differences in *E. coli* concentrations between the canal and Amstel River locations were not statistically significant (*p* > 0.05). Notably, *E. coli* concentrations above 5.0 log_10_ copies/100 mL were occasionally detected at CANAL 1 in August and CANAL 2 in June, suggesting an incidental high input of fecal contamination.

*Campylobacter* was also detected at all locations throughout the sampling period, exhibiting a wide variance in concentration ranging from 1.7 to 6.4 log_10_ copies/100 mL (Fig. 3). Higher concentrations of *Campylobacter* were consistently observed at CANAL 3, CANAL 2, and CANAL 1 compared to the Amstel River locations, with the highest concentrations observed at CANAL 3 in June and July (6.4 and 5.7 log_10_ copies/100 mL, respectively). The results from the ANOVA test showed that *Campylobacter* concentrations were significantly different between locations (*p* < 0.01), with CANAL 3 having significantly higher concentrations than CANAL 2 (*p* < 0.05) and the different Amstel River locations (*p* < 0.01, Table S3).

Adenovirus was detected in 17 out of 36 samples, with concentrations ranging from 0.2 to 2.6 log_10_ copies/100 mL in the positive samples (Fig. 3). Significantly higher adenovirus concentrations were observed at all locations in October (ranging from 1.7 to 2.6 log_10_ copies/100 mL) compared to the other months (*p* < 0.05, Table S3), except for August. Furthermore, adenovirus was detected at similar frequencies in CANAL 1, CANAL 2, and AMSTEL 3, while the highest concentrations were observed at AMSTEL 2; however, concentrations at this site were not statistically different from those at other locations (*p* > 0.05, Table S3).

*Cryptosporidium* was only detected once, at CANAL 1 in August with a concentration of 1.8 log_10_ copies/100 mL. *Giardia B* was also only detected once, at AMSTEL 1 in May with a concentration of 1.4 log_10_ copies/100 mL.

#### Non-fecal microorganisms

*P. aeruginosa* was detected in most samples (30 out of 36) and exhibited a relatively small variance in concentrations that ranged from 1.1 to 2.7 log_10_ copies/100 mL (Fig. 3). There was, however, a noticeable difference in the frequency of positive detections both spatially and temporally. Spatially, *P. aeruginosa* was consistently detected each month samples were taken at locations AMSTEL 3, CANAL 1, and CANAL 2, whereas at other locations three to five of the six samples were positive during the six month monitoring period. Temporally, *P. aeruginosa* was detected across all six locations in May, September, and October, and it was detected at less than three locations in the other three months.

*L. pneumophila* was detected in 14 out of 36 samples in a concentration ranging from 2.1 to 3.5 log_10_ copies/100 mL (Fig. 3). *L. pneumophila* was not evenly detected across different locations or monitoring months. The highest frequencies (five out of six samples) were found at the location AMSTEL 2 or in October (five of the six locations). In October, *L. pneumophila* showed also the highest concentrations, ranging from 2.9 to 3.6 log_10_ copies/100 mL, compared to the other sampling months. These *L. pneumophila* concentrations were, however, not statistically different from the other months (*p* > 0.05, Table S3).

*Leptospira* was detected in 16 out of 36 samples, with concentrations ranging from 0.3 to 1.8 log copies/100 mL (Fig. 3). Spatially, *Leptospira* was more frequently detected at locations CANAL 2 and CANAL 1 compared to the other locations. Temporally, *Leptospira* was most prevalent in May, with positive detections at five of the six sampling locations. The highest concentrations, ranging from 1.3 to 1.8 log_10_ copies/100 mL at each location, were also observed in May, although these differences were not statistically significant compared to other months (*p* > 0.05, SI2, Table S3).

### Water temperature and rainfall

The total weekly rainfall preceding the sampling day was highest in October (42.2 mm), followed by July (8.8 mm) (Table 2). The other four months experienced minimal rainfall (non to 0.12 mm). The lowest and highest water temperatures recorded were 15.0 °C in October and 24.8 °C in September, respectively. The water temperature in October was significantly lower than in September, August, or June (*p* < 0.05, Table 2, SI2, Table S3). In addition, the water temperature in May was also significantly lower than in September (*p* < 0.05).

**Table 2:** Water temperature at each location from each sampling month and rainfall (total precipitation in the 7 days before the sampling date)

	**May**^**#**^	June*	**July**	August*	September*^#^	October*		
**AMSTEL1**	18.2	23.8	22.3	21.0	24.8	15.0	**Water temperature/°C**	
**AMSTEL2**	19.4	22.6	21.2	22.8	24.6	15.4		
**AMSTEL3**	19.2	22.7	21.8	22.6	24.4	14.6		
**CANAL1**	19.0	23.0	22.0	22.7	24.6	15.1		
**CANAL2**	20.6	22.9	21.7	22.9	23.9	15.1		
**CANAL3**	18.6	22.9	21.7	22.9	24.0	15.2		
	0.0	0.0	8.8	0.1	0.0	42.2	**Rainfall/mm**	

^*,#^Water temperature at each month show significant difference at 0.05 level

### Correlations between microbial parameters and microbial to water temperature and rainfall

A significant positive correlation of fecal and non-fecal pathogens to *E. coli* was only observed for *Campylobacter* (*p* < 0.01, rho = 0.50). Adenovirus,* P. aeruginosa*,* L. pneumophila*, and *Leptospira* concentrations in the water samples from the Amsterdam surface water system were not significantly correlated to *E. coli* (*p* > 0.05; Table 3). In addition, a significant positive correlation between the non-fecal pathogen *L. pneumophila* and the fecal pathogen adenovirus was observed (*p* < 0.01, rho = 0.44) (Table 3).

**Table 3:** Spearman’s correlation between concentrations of pathogens and water temperature, and rainfall

	***E. coli***	***Campylobacter***	**Adenovirus**	***P. aeruginosa***	***L. pneumophila***	***Leptospira***	**Rainfall**	**Water temperature**
*E. coli*	1	0.50^**^	0.29	0.26	0.07	−0.02	0.04	−0.15
*Campylobacter*	0.50^**^	1	0.07	0.13	−0.04	0.13	0.3	−0.08
Adenovirus	0.29	0.07	1	0.01	0.44^**^	−0.22	0.57^**^	−0.50^**^
*P. aeruginosa*	0.26	0.13	0.01	1	−0.23	0.19	−0.33	0.29
*L. pneumophila*	0.07	−0.04	.44^**^	−0.23	1	−0.11	0.35*	−0.42*
*Leptospira*	−0.02	0.13	−0.22	0.19	−0.11	1	−0.14	−0.07
Rainfall	0.04	0.3	0.57^**^	−0.33	0.35*	−0.14	1	−0.60^**^
Water temperature	−0.15	−0.08	−0.50^**^	0.29	−0.42	−0.07	−0.60^**^	1

^*^Correlation is significant at the 0.05 level (2-tailed)

^**^Correlation is significant at the 0.01 level (2-tailed)

The results from the correlation analysis between pathogen concentrations and water temperature and rainfall showed significant correlations between adenovirus and both rainfall and water temperature (*p* < 0.01) (Table 3). The correlation between adenovirus concentrations and rainfall was positive (rho = 0.57) (Table 3), and stronger than the negative correlation of adenovirus concentrations with water temperature (rho = −0.50) (Table 3). A significant negative correlation between rainfall and water temperature was observed as well (*p* < 0.01) (Table 3).

## Discussion

### Spatial distribution of *E. coli* and fecal and non-fecal pathogens in urban surface water

The broad distribution and variable concentrations of *E. coli* and *Campylobacter* in the Amstel River and inner canals of Amsterdam from May to October 2023 (Fig. 3) indicate that the urban surface water is fecal contaminated, including gastroenteritis pathogens. The stronger spatial variability of *Campylobacter* compared to *E. coli* likely reflects localized contamination sources, whereas *E. coli* originates from multiple diffuse inputs. As we used qPCR to target the heat shock protein 70 (hsp 70) or 16S rRNA gene of *E. coli* and *Campylobacter* respectively, it is not possible to directly compare our data with the EU Bathing Water Directive 2006/7/EC concerning bathing water quality, which is based on *E*. coli and intestinal enterococci concentrations using culturing methods (European Parliament and Council of the European Union, 2006). Consequently, the present study was not intended to evaluate regulatory compliance. Instead, the qPCR-based monthly spatiotemporal monitoring approach was used to investigate contamination dynamics and co-occurrence patterns of fecal indicators and pathogen-associated markers in Amsterdam urban surface waters and investigate how well *E. coli* is an indicator for these fecal and opportunistic pathogens. However, a previous study showed that *E. coli,* quantified using culturing methods, generally exceeded the standards for good bathing water quality in the urban water system of Amsterdam in 2012 (Sales-Ortells et al., 2015). Our results, using qPCR, demonstrates that *E. coli* is still present at relatively high concentrations in the Amsterdam surface water in 2023. Furthermore, *Campylobacter* concentrations in our study are comparable to those reported in Amsterdam by Sales-Ortells et al. (2015), who also used qPCR targeting the same gene (Sales-Ortells et al., 2015).

Spatial differences in microbial water quality across the sampling locations in Amsterdam were observed. For example, at location AMSTEL 1, where the Amstel River enters the city of Amsterdam, we consistently detected *E. coli* and *Campylobacter* and occasionally adenovirus and *Giardia B* only once (Fig. 3). This location is nearby the Rondehoep polder, where meadow birds and waterfowls reside, known sources for *E. coli* and *Campylobacter*. Furthermore, cattle grazes in this polder, which can harbor *Giardia B* (Thompson & Monis, 2012). Based on these results, we hypothesize that the Rondehoep polder could be responsible for part of the fecal contamination at sampling location AMSTEL 1. In a previous monitoring study on microbial water quality in Amsterdam, *Giardia* was reported within the range of 8–157 cysts/10 L in Amstel River which is near to the location AMSTEL 3 in this study (Schets et al., 2008). The source of *Giardia* contamination in that study remained unclear. However, compared to our findings, the lower detection frequency in 2023 compared to 2004 near AMSTEL 3 may reflect differences in sampling periods, environmental conditions, and methodological approaches between studies, rather than a definitive reduction in contamination over time.

We expected to observe a clear influence of the WWTP effluent discharge on FIB concentrations at AMSTEL 2, but this was not observed. The *E. coli* and *Campylobacter* concentrations did not increase at AMSTEL 2 compared to AMSTEL 1 (Fig. 3). Instead, the highest detection frequency for adenovirus was observed at AMSTEL 2 (Fig. 3), indicating human fecal contamination from the WWTP effluent, although this cannot be confirmed without human-specific microbial source tracking markers. We speculate that the flow of the WWTP effluent through the polder water area prior to entering the Amstel River (Sales-Ortells et al., 2015) could explain the low contribution of WWTP effluent to the *E. coli* and *Campylobacter* concentrations at AMSTEL 2. Adenovirus is more resistant to environmental conditions, such as UV or high temperature, than *E. coli* or *Campylobacter* due to the non-enveloped structure of adenovirus (Bofill-Mas et al., 2010). In contrast, cell membrane integrity of *E. coli* and *Campylobacter* could have been compromised by the environmental conditions during the WWTP effluent flow through the polder, resulting in released DNA, preventing detection with qPCR after filtration. This possible difference in die-off patterns may, thus, explain why adenovirus was detected more frequently, but not for *E. coli* and *Campylobacter*, from the wastewater effluent into the Amstel River. It is important to recognize that persistence of microbial genetic material in environmental waters may differ substantially from the persistence of viable bacteria, infectious viruses, or culturable organisms. Environmental decay rates vary among microbial targets and between molecular and culture-based detection approaches, potentially resulting in prolonged detection of nucleic acids after loss of infectivity or culturability (Boehm et al., 2019, 2022). Therefore, qPCR-based detection should be interpreted as evidence of contamination signatures and potential pathogen occurrence rather than direct evidence of infectious risk or regulatory exceedance.

*L. pneumophila* was also frequently detected at AMSTEL 2 (Fig. 3), which seems surprising, since *L. pneumophila* is a non-fecal pathogen. However, several studies have shown that *L. pneumophila* can multiply in certain biological treatment processes of wastewater treatment plants (Caicedo et al., 2019), also in the Netherlands (van den Berg et al., 2023). The correlation between adenovirus and *L. pneumophila* at this location (Table 3) may suggest that *L. pneumophila* also have originated from the wastewater effluent. The lack of correlation between *L. pneumophila* and *E. coli* at this location (Table 3) indicates that both bacteria behave differently in surface water. Previous studies have shown that *E. coli* typically decays relatively rapidly in surface waters due to environmental stressors such as UV radiation and temperature fluctuations, whereas adenoviruses are more persistent because of their non-enveloped structure (Bofill-Mas et al., 2010; Korajkic et al., 2018). In contrast, *L. pneumophila* exhibits distinct environmental behavior, including persistence within biofilms and protozoan hosts, although its growth in surface water is generally limited at lower temperatures (National Academies of Sciences, Engineering, and Medicine, 2019; van der Wielen et al., 2023).

Interestingly, *P. aeruginosa* was detected with a higher frequency at AMSTEL 3 than at AMSTEL 1 and AMSTEL 2 (Fig. 3). However, given the limited sample size per location (*n* = 6), this difference was not statistically evaluated and should be interpreted with caution. The presence of *P. aeruginosa* at AMSTEL 3 may be related to recreational activities in this area, as human shedding has been reported as a potential source of *P. aeruginosa* in recreational waters (Januário et al., 2019). At the same time, exposure to *P. aeruginosa* may also occur directly from the water itself, and thus, the direction of transmission cannot be determined from this study. In addition, also at CANAL 1 and CANAL 2, *P. aeruginosa* was occasionally detected in high concentrations. A wide distribution of *P. aeruginosa* in marine water fresh water and sewage, and the ability to grow at a low level of nutrients and in a wide temperature range (15–30 °C) were also reported from previous studies (Januário et al., 2019; Mohammed et al., 2012; van der Wielen et al., 2023). However, a relation between *P. aeruginosa* concentrations and the water temperature was not observed in our study (Table 3), which may partly reflect the limited number of samples and other unknown factors affect the persistence and/or growth of *P. aeruginosa* in these urban surface waters. As *P. aeruginosa* concentrations in the Amsterdam urban water system did not correlate to the fecal bacteria and did not always relate to wastewater sources, the exact source of *P. aeruginosa* in urban surface water, especially at the inner canals in Amsterdam, or the exact conditions that enhance growth of the organism remain unknown.

The inner canals generally showed higher concentrations of fecal-associated microorganisms compared to the Amstel River locations. *Campylobacter* concentrations were higher at all inner canal locations than in the Amstel River (Fig. 3). The elevated fecal bacterial concentrations observed in the inner canals may reflect contributions from multiple potential sources, including houseboats, waterfowl, and combined sewer overflows during rainfall events, although the relative importance of these sources could not be determined in this study (Schets et al., 2008). Furthermore, *Leptospira* was also frequently detected, especially at CANAL 1 and CANAL 2 (Fig. 3), indicating possible contamination through urine from brown rats, rodents and/or dogs in the surface water (Becker et al., 2017; Pijnacker et al., 2016; Richard et al., 2022). Pathogenic *Leptospira* were detected in 35% of bathing water samples in the Netherlands during a screening study in 2017 (Becker et al., 2017). These observations may suggest a potentially higher exposure to *Leptospira* in the inner canals compared to other locations, although direct health risk assessment was outside the scope of this study.

### Temporal distribution of pathogens in surface water

The results obtained indicate that weather conditions influenced the temporal distribution of pathogens in Amsterdam. The most striking seasonal observation from our study was the higher concentrations of human adenovirus and *L. pneumophila* in October compared to the other months (Fig. 3), and which coincide with heavy rainfall (Table 2). These findings are generally consistent with our assumption that rainfall events result in increased pathogen loads due to run-off, although the observed relationships are influenced by a limited number of rainfall events. However, others could not detect adenovirus 40/41 in a rain harvest sedimentation pond and a wadi in Amsterdam when daily monitored during heavy rainfall (Sales-Ortells et al., 2015). This difference might have been caused by the higher detection limit for adenovirus 40/41 in that study compared to ours, since we sampled 50 L whereas Sales-Ortells et al. (2015) sampled only 1 L. The concentrations of adenovirus 40/41 we detected ranged from 1 to 370 copies/100 mL, which was below or near the detection limit in the study by Sales-Ortells et al. (2015). Others reported pathogen resuspension from sediment into the overlaying river water during rainfall periods, although the contribution of this resuspension to the total pathogen concentration in the river water could not be established, as other sources (e.g. run-off) also contributed to the increased pathogen concentration (Wu et al., 2009). It is likely that resuspension and contribution from other sources during heavy rainfall also contributed to the higher human adenovirus and *L. pneumophila* concentrations in the Amsterdam surface water. Haramoto et al. (2006) observed 1.6 log units increase of human adenovirus in seawater in Japan one day after an 84.5 mm heavy rainfall event, which was explained by the input from untreated sewage water or combined sewer overflow (Haramoto et al., 2006). Based on these studies, the elevated adenovirus concentrations observed during the heavy rainfall period in October 2023 are consistent with potential contributions from multiple hydrological processes, including increased surface runoff, combined sewer overflows, and sediment resuspension. However, the relative contribution of these mechanisms could not be determined in this study because hydrological flow data, sewer overflow records, and sediment measurements were not available.

The higher concentrations of *L. pneumophila* in October were also significantly positively correlated with rainfall (Fig. 3, Table 3, *p* < 0.05). *L. pneumophila* was detected with a mean concentration of 2.49 log_10_ copies/100 mL in a sedimentation pond in Amsterdam, which increased slightly to 2.94 log_10_ copies/100 mL after heavy rainfall (Sales-Ortells et al., 2015). Similarly, *L. pneumophila* has also been observed in rainwater puddles on roads and an epidemiological relationship between legionellosis and heavy rainfall has been observed as well (Fisman et al., 2005; Hicks et al., 2007; Sakamoto et al., 2009). Therefore, the observed increase in *L. pneumophila* concentrations following heavy rainfall suggests a potential increase in exposure risk during recreational activities involving aerosol formation (e.g., splashing, aspiration, fountains). However, the underlying mechanisms remain uncertain and may involve combined influences of runoff, wastewater-related inputs, and sediment-associated release processes.

In contrast to adenovirus and *L. pneumophila,* no significant increase in *E. coli* and *Campylobacter* was observed following rainfall (Fig. 3; Table 3). As discussed above, the greater environmental persistence of adenovirus and *L. pneumophila* may partly explain this difference. Additional contributing factors may include inputs from wastewater treatment plant effluent and other sources, such as sediment resuspension along the Amstel River (Wu et al., 2009). In the inner canals, runoff and sewer overflows may represent important sources of adenovirus and *L. pneumophila* (Haramoto et al., 2006), whereas for *E. coli* and *Campylobacter*, runoff and sewer overflows are likely dominant contributors following heavy rainfall events (Sales-Ortells et al., 2015). Because the study relied on monthly sampling and city-scale rainfall data, the relative influence of seasonal variation, temperature, runoff, sewer overflow events, and sediment resuspension on pathogen occurrence could not be fully separated. In addition, although lower water temperatures in October may have contributed to enhanced microbial persistence, this mechanism alone is unlikely to explain the observed patterns, as no comparable rainfall-associated increase was detected for *E. coli* or *Campylobacter*.

### *E. coli* as indicator organism for fecal and non-fecal pathogens and implications for monitoring

In our study, a weak positive Spearman correlation was observed between *Campylobacter* and *E. coli* (Table 3), and additionally, the concentrations of both pathogens are comparable with each other (Fig. 3), indicating that during the monitoring period of our study *E. coli* could be a reasonable indicator for the fecal pathogen *Campylobacter* in the Amsterdam urban water system. Jones (2001) summarized studies where correlation between *Campylobacter* and FIB in surface waters were studied. Some studies showed a weak or no correlation between *Campylobacter* and FIB in surface water, whereas other studies showed good correlations. It was concluded that when the FIB and *Campylobacter* came from different sources (e.g., FIB from WWTP effluent and *Campylobacter* from waterfowl) correlations were weak. Given the mixed sources in the Amsterdam urban water system and the moderate strength of the correlation observed in this study, *E. coli* should be interpreted with caution as an indicator for *Campylobacter*, and no definitive conclusions regarding shared sources can be drawn. Site-specific analyses could further clarify source contributions but were not feasible due to limited sample size.

The abundance of the fecal pathogen adenovirus was not significantly correlated with *E. coli*, partly because adenovirus was only occasionally detected above the detection limit, whereas *E. coli* was always observed. Furthermore, adenovirus and *E. coli* showed different occurrence patterns, with adenovirus being intermittently detected and not correlated with *E. coli*, in contrast to the consistent detection of *E. coli* across all samples. For example, adenovirus concentrations were highest at AMSTEL 2, whereas *E. coli* levels were not elevated at that location compared to the other location (Fig. 3). This finding is not surprising, as others also did not observe significant correlations between *E. coli* and adenovirus (Bofill-Mas et al., 2010; Sales-Ortells et al., 2015). It was reported that the correlations in persistence between *E. coli* and viruses like adenovirus in surface water is weak, because adenovirus have a higher resistance to environmental conditions than *E. coli* (Korajkic et al., 2018). This difference in survival behavior can be the reason why higher adenovirus concentrations were observed at the location downstream of the WWTP effluent discharge point, whereas *E. coli* concentrations were not elevated at that point. Another possible reason why adenovirus and *E. coli* concentrations in the Amsterdam urban surface water were not correlated is that *E. coli* can also come from non-human fecal sources, whereas adenovirus 40/41 always comes specifically from human sources. The use of microbial source tracking markers, such as human-associated *Bacteroides* (e.g., HF183), could be an option for future studies to better distinguish contamination sources in urban surface waters (Harwood et al., 2014).

Korajkic et al. (2018) conducted a comprehensive review examining the correlation between fecal indicators and pathogens in recreational water. Their findings indicated that in six out of 41 studies, *E. coli* correlated significantly with *Cryptosporidium/Giardia (oo)cysts*, in four out of 41 studies with *Campylobacter* spp.*,* and in two out of 41 studies with adenoviruses. These authors concluded that frequency of detection, difference in fate and transport characteristics and differences in broad site-specific factors, such as the sources of pathogens, were the reasons why *E. coli* did not significantly correlate with the fecal pathogens in most of these studies. As indicated above, these factors are also the likely explanation that, apart from *E. coli* and *Campylobacter,* we did not observe significant correlations between *E. coli* and fecal pathogens (Table 3). Besides, we also determined that *E. coli* did not significantly correlate with any of the non-fecal pathogens detected in this study (Table 3). It is well-established that fecal and non-fecal bacteria show distinct behaviors in surface water regarding survival and growth, and bacteria originating from water sources are also with significant variability in growth rates and resilience to environmental conditions (Bogosian & Bourneuf, 2001). The lack of a correlation between most pathogens analyzed in our study confirms previous studies that it is challenging, if not impossible, to identify a single indicator organism that can predict the abundance and fate of all pathogens. Consequently, to more reliable identify health risks related to surface water recreation, it might be necessary to monitor multiple indicator organisms or even the most important pathogens. However, the detection and enumeration of pathogens in surface water are analytically challenging due to their low concentrations and often require large sampling volumes, which may limit routine application. In this context, microbial source tracking markers could provide a practical complementary approach, as they are typically more abundant and easier to quantify during contamination events; however, they do not directly reflect pathogen concentrations or health risks and may differ in environmental persistence compared to pathogens (Harwood et al., 2014; Korajkic et al., 2018).

Based on our findings, the following points are suggested to include in future monitoring in Amsterdam. Human adenovirus and *L. pneumophila* are more likely to enter surface waters after heavy rainfall and near wastewater treatment plant discharges. More frequent monitoring of these pathogens is recommended, especially in areas close to sewage inputs. For *E. coli*, *Campylobacter*, and *Leptospira*, regular and more frequent monitoring in inner canals of Amsterdam, supplemented with suitable human or animal source tracking markers, where there is high human, dog and rat activity, will help better assess these pathogen levels. These findings suggest that *E. coli* monitoring alone may not fully capture the diversity of microbial hazards present in urban surface waters, particularly for environmentally persistent non-fecal pathogens. Consequently, including representative organisms for non-fecal pathogens in routine monitoring might be needed to warn the public of the presence of these pathogens in recreational surface water sites (Gao et al., 2024; Januário et al., 2019; Korajkic et al., 2018).

## Conclusions

This study investigated the occurrence of *E. coli*, four fecal pathogens (*Campylobacter*, human adenovirus, *Cryptosporidium*, and *Giardia*) and three non-fecal pathogens (*P. aeruginosa*, *L. pneumophila*, and *Leptospira*) in 36 surface water samples from the Amstel River and inner canals of Amsterdam. Water temperature and rainfall data were collected to explore potential correlations with pathogen concentrations. By selecting different sampling locations along the Amstel River and inner canals in Amsterdam during May to October 2023, we aimed to determine the spatiotemporal dynamics of these organisms in the Amsterdam surface water system and investigated how the measured parameters were related with each other.

The results indicate that microbial water quality in the Amsterdam surface water system is influenced by multiple contamination sources, leading to spatial and temporal variability in pathogen occurrence. This variability highlights the complexity of urban water systems and the limitations of relying on a single indicator organism to represent diverse microbial risks.

Notably, the frequent detection of opportunistic pathogens such as *P. aeruginosa* indicates that recreational health risks extend beyond fecal contamination to include environmentally persistent microorganisms.

Wastewater treatment plant (WWTP) effluent was identified as an important contributor to the presence of human adenovirus and *L. pneumophila* in the Amstel River, with rainfall events further amplifying their concentrations, likely through increased runoff and sewer overflows. In contrast, elevated *Leptospira* concentrations in the inner canals were associated with densely populated urban areas, suggesting a potential link to higher densities of animal reservoirs such as rodents and domestic animals.

Importantly, *E. coli* showed limited correlation with most of the investigated fecal and non-fecal pathogens, indicating that fecal indicator bacteria alone are insufficient to capture the diversity and dynamics of microbial hazards in urban surface waters. These findings highlight the need for more comprehensive monitoring strategies that incorporate multiple microbial targets and environmental drivers to better inform public health risk assessments in urban aquatic systems.

Because qPCR-based detection does not distinguish between viable and non-viable organisms, the reported concentrations represent potential occurrence and exposure indicators rather than direct measures of infectivity. In addition, several environmental and hydrodynamic parameters that may influence pathogen transport and persistence, including flow conditions, water mixing, residence time, and timing of combined sewer overflow events, were not measured in this study. In addition, rainfall data were obtained from a city-scale weather database and may not fully capture localized storm heterogeneity or site-specific runoff dynamics. Consequently, the observed associations between rainfall and pathogen occurrence should be interpreted cautiously, and direct mechanistic attribution to specific hydrological processes was beyond the scope of this study.

## Supplementary Information

Below is the link to the electronic supplementary material.

ESM 1 (PDF 195 KB)

ESM 2 (XLSX 21.7 KB)

## Data Availability

No datasets were generated or analysed during the current study.
