# Supplementary material for: Spatiotemporal distribution of Escherichia coli and fecal and non-fecal pathogens in the urban surface water system of Amsterdam
Source: Environ Monit Assess. 2026 Jul 20;198(8):861. doi: 10.1007/s10661-026-15653-y (PMC13385234; doi:10.1007/s10661-026-15653-y)
Supplement: Supplementary file 1 — (PDF 195 KB) [file 10661_2026_15653_MOESM1_ESM.pdf]

Spatiotemporal distribution of *Escherichia coli*, and fecal and non-fecal pathogens in the urban surface water system of Amsterdam

Environmental Monitoring and Assessment

Sha Gao [a], Thomas Wagner [a], Paul van der Wielen [b], Huub Rijnaarts [a], Nicolae Șișcanu [a], Nora Sutton [a*]

[a] Department of Environmental Technology, Wageningen University, PO Box 17, 6700 EV Wageningen, The Netherlands

[b] KWR Water Research Institute, Groningenhaven 7, 3433PE Nieuwegein, The Netherlands

* Corresponding Author: nora.sutton@wur.nl; tel. +31 (0)317 483339

Supplementary information 1(SI1)

S1 Calculation of the copy numbers of microorganisms in 50 L water samples.

DNA extraction efficiency in sludge and supernatant from centrifugation of each sample was calculated by equation S2.1:

$$R = \frac{SQ_{Samples\ IC}}{SQ_{\ IC}} * D * 100\% \qquad \text{Eq S2.1}$$

R: DNA extraction efficiency;

$SQ_{SampleIC}$: starting quantity of IC from qPCR in DNA samples added at the 1st step of DNA extraction;

$SQ_{IC:}$ starting quantity of IC from qPCR;

D: dilution factor of IC in samples.

Copy numbers of each microorganism in Sludge was calculated by equation S2.2:

$$C_{Sludge} = \frac{SQ_m}{V_{qPCR}} * \frac{V_D}{R} * \frac{V_S}{V_{SD}} \qquad \text{Eq S2.2}$$

C_{Sludge}: copy numbers of microorganisms in sludge; unit in copy numbers/0.5mL;

SQ_m: starting quantity of microorganisms from qPCR;

V_{qPCR}: volume of DNA template for qPCR;

V_D: volume of DNA samples from sludge;

R: DNA extraction efficiency;

V_S: volume of sludge samples from the centrifugation; 0.5 mL in this study;

V_{SD}: volume of sludge samples for DNA extraction.

Copy numbers of each microorganism in Supernatant was calculated by equation S2.3:

$$C_{Supernatant} = \frac{SQ_m}{V_{qPCR}} * \frac{V_{D1}}{R} * \frac{V_{sup}}{V_F} \qquad \text{Eq S2.3}$$

$C_{Supernatant}$: copy numbers of microorganisms in supernatant; unit in copy numbers/1.495L;

SQ_m: starting quantity of microorganisms from qPCR;

V_{qPCR}: volume of DNA template for qPCR;

V_{D1}: volume of DNA samples from supernatant;

R: DNA extraction efficiency;

V_{sup}: volume of supernatant samples from the centrifugation; 1.495 L in this study;

V_F: volume of supernatant samples filtrate on membrane for DNA extraction.

Copy numbers of each microorganism in 50 L water sample was calculated by equation S2.4:

$$C = \frac{C_{Supernatant} + C_{Sludge}}{50\% * 500} \qquad \text{Eq S2.4}$$

C: copy number in 50L water samples; unite in copy numbers/100mL.

50%: the recovery from Hemoflow filtration system
